# Cellular Immunology and COVID-19

**DOI:** 10.3390/cells10123591

**Published:** 2021-12-20

**Authors:** Isabella Quinti

**Affiliations:** Department of Molecular Medicine, Sapienza University of Rome, 00185 Rome, Italy; isabella.quinti@uniroma1.it

In “Cellular Immunology and COVID-19” (a Special Issue of *Cells*), a panel of leading scientists provides an exhaustive overview of the different aspects of the immune mechanisms underlying COVID-19. Collectively, the 17 contributions in “Cellular Immunology and COVID-19” highlight the main molecular and therapeutic aspects of inflammation, innate immunity and adaptive immunity in SARS-CoV-2 infection with the suggestion of possible therapeutic implications. The success of the policy of vaccinating the population en masse to contain the coronavirus disease 2019 (COVID-19) pandemic still requires extensive work. Basic science strongly contributed to the understanding of the role played by a variety of immune cell types, but SARS-CoV-2 infection is still challenging the scientific community. Both innate and adaptive immune cells’ alterations at the infection’s onset and during and after the course of COVID-19 have been extensively described. Effective vaccines against SARS-CoV-2 are being administered worldwide, with the aim of terminating the COVID-19 pandemic. As for all the immunizations, the efficacy has been linked to the production of specific antibodies, which increase in response to all vaccines in use. A comparison between immune mechanisms during infection and after immunization among persons vaccinated at different times offers the possibility to dissect the difference between the immune response to a natural course of infection, primarily characterized by the function of the innate immune system, with a secondary involvement of T and B cells, and SARS-CoV-2 vaccines designed to force the adaptive immune system to generate adaptive immune responses. The innate system mechanisms have been addressed in seven papers. In their review article, Bahareh Hafezi and colleagues describe the role of mast cells as key effector cells of the innate immune system during viral infections. The activation of mast cells, located in the submucosa of the respiratory tract, is known to lead to the release of pro-inflammatory cytokines, contributing to inappropriate antiviral immune responses. However, often overshadowed by their involvement in pathology, mast cells play an important role in protective immunity as multi-equipped pathogen sensors. Furthermore, mast cell activators might act as adjuvants for COVID-19 vaccines, and the drugs targeting the functions of mast cells could be of value in the treatment of COVID-19 [1]. Viral infections can alter the host miRNA expression, impair signaling pathways, modulate host–virus interactions, regulate viral infectivity and transmission, and result in the differential activation of antiviral immune responses. Irma Saulle et al. summarized the findings related to an in utero mother-to-child SARS-CoV-2 infection associated with a generalized hyper-activation of the immune response causing the alteration of the immunological barrier. A number of molecular mechanisms, among which microRNAs have recently emerged, played a leading role. The synthesis of miRNAs coupled with a release of pro-inflammatory cytokines/chemokines and type I interferons at the placental level seems to control both the infection and the dysfunctional immune reaction, thus representing a positive correlate of protection and a potential therapeutic target against SARS-CoV-2 [2].

Over the course of COVID-19, neutrophilia is a predictor of a poor outcome, and the changes in neutrophils’ activation and degranulation indicates their dysfunctionality. An elevated secretion of neutrophil extracellular traps (NETs) levels was found, and plasma NETs correlated with the severity of the disease by interacting with T cells [3]. Iwona Kwiecień et al. described new neutrophil activation parameters assessing maturation, reactivity, granularity and the neutrophil volume that is able to distinguish convalescent patients from patients with an active SARS-CoV-2 infection and healthy controls [4]. The expansion of myeloid-derived suppressor cells (MDSC) has been described in severe COVID-19. MDSCs in humans are polymorph nuclear MDSCs, or monocyte MDSCs, both having the ability to reduce inflammation by suppressing innate and adaptive immune function through several mechanisms, including iNOS, Arg-1, nicotinamide adenine dinucleotide phosphate oxidase (NOX2) and transforming growth factor beta, as detailed in the paper by Alessandra Sacchi and colleagues. Early after SARS-CoV-2, the MDSCs population produces arginase I and nitric oxide synthase, detracting the microenvironment from arginine, inducing platelet activation, impairing nitric oxide synthesis, expanding infection and predicting the fatal outcome of the disease. Moreover, PMN-MDSC from COVID-19 patients, being able to increase platelet activation by reducing the L-arginine concentration, contributed to platelet hyperactivity, causing massive platelet activation and thrombotic events in severe COVID-19 cases. In addition, the expression of SARS-CoV-2 receptor ACE-2 on platelet membranes suggests a possible direct role of SARS-CoV-2 in platelet activation [5]. A higher proportion of low-density granulocytes in severe and critical COVID-19 and their capacity to produce NETs correlated with severity and inflammatory markers. As defined by Jiram Torres-Ruiz et al., the sera from severe/critical COVID-19 patients had a lower degradation capacity of NETs and anti-NET antibodies and were related to the presence of ANA and ANCA autoantibodies [6]. Moreover, the cytokine hyper activation in COVID-19 appears to be similar to that seen in rheumatoid arthritis, shedding light on the pathological crosstalk between COVID-19 and rheumatoid arthritis, and the aspects of SARS-CoV-2 infection in RA development [7].

Four papers addressed new aspects of the role of T cells. In their research paper, Diana Martonik and co-authors describe the contribution of the dysregulation of the Treg/Th17 cells ratio skewing towards the Th17 phenotype for the uncontrolled release of cytokine and chemokine cascades in COVID-19 patients, leading to aggravated inflammatory responses and tissue damage. IL-17 might promote pulmonary inflammation, following an infection by neutrophil and monocyte migration to the lungs, and by activating other cytokine cascades (G-CSF, TNFα, IL-1β and IL-6). Currently, there are several biological drugs targeting IL-17 and IL-23 that have been approved for the treatment of rheumatologic diseases, and there are biological drugs that intervene in the cell differentiation toward the Th17 phenotype through blocking STAT3 [8]. Juliana G. Melgaço et al. proposed a model to evaluate, in vitro, the events induced by a viral protein, or whole virus SARS-CoV-2. T-cell recognition, activation and, consequently, IFN-gamma gene expression, IFN-gamma production by CD4+ T cells and the effects of immune response products in a lung cell line in the presence of SARS-CoV-2 might be tested. In addition, the model allows for the analysis of the positive effects on apoptosis reduction in alveolar cells infected by the new coronavirus using the product of virus-specific immune response by people who were exposed previously to SARS-CoV-2, thus suggesting that a second exposure might collaborate with a strong immune response [9]. T cells, especially the cytotoxic CD8+ T cells, are critical for viral recognition and clearance. The X-ray crystallography structure of a TCR shed light on the molecular basis of a public TCR, recognizing a dominant spike-derived SARS-CoV-2 epitope. A dominant spike-derived CD8+ T cell epitope activates a poly functional CD8+ T cell response in recovered COVID-19 patients, possibly being a promising target to prime and boost CD8+ T cells against SARS-CoV-2 infection [10]. Assia Eljaafari et al. summarize the current knowledge on the PD-L1/PD-1 immune checkpoint axis as the strongest T cell exhaustion inducer. PD-L1/PD-1 expression is overexpressed in the white adipose tissue of obese individuals during IFNγ secretion, leading to T cell dysfunction and notably reduced cytolytic activity, shedding light on why adipose-tissue-infiltrating viruses, such as SARS-CoV-2, can worsen disease in obese individuals [11].

The kinetics of neutralizing antibodies (NAbs) and anti-SARS-CoV-2 anti-S-RBD (receptor-binding domain) IgGs has been extensively analyzed in the recent literature with a progressive increase in knowledge. Two papers by Evangelos Terpos et al. and by Andrzej Tretyn and colleagues described the decline in specific humoral immunity showing persistent but declining anti-SARS-CoV-2 antibody responses following full vaccination, with NAbs and anti-S-RBD having a rapid increase at a constant rate of about 3% per day during the firsts weeks after immunization but a lower decline rate later on [12,13]. However, while specific antibody levels decline two months after the second vaccine dose, highly specific memory B cells continue to increase, thus predicting sustained protection from COVID-19, as shown by Eva Piano Mortari et al. However, the vaccine does not induce sterilizing immunity, since mucosal IgA is not induced by the vaccination, and infection after vaccination may be caused by the lack of local preventive immunity because of the absence of mucosal IgA [14]. Additional data provided information on the antibody responses in patients with a prior COVID-19 infection. Pulvirenti F. and colleagues showed that COVID-19 was associated with superior antibody responses over time in healthy donors and in patient populations such as patients with primary antibody deficiencies. Spike-specific IgG was induced more frequently after infection than after vaccination, and the antibody response was boosted in convalescents by vaccination. Although immunized patients with primary antibody deficiency generated atypical memory B cells, possibly by extra-follicular or incomplete germinal center reactions, SARS-CoV-2 convalescents responded to the infection by generating spike-specific memory B cells that were improved by the subsequent immunization. Thus, natural infection responses were boosted by a subsequent immunization, suggesting the possibility of further stimulating the immune response by additional vaccine doses [15].

In addition, the role of training immunity was described by Espiridión Ramos-Martinez et al., demonstrating that revaccination with BCG in persons occupationally exposed to COVID-19 increased serum cytokine concentrations and neutralizing antibody titers, and this was synergized with a subsequent vaccination against SARS-CoV-2 [16].

Finally, we are all aware of the challenges due to the development and evolution of therapeutically resistant strains, including those belonging to a variant of concern (VOC) and a variant of high consequence (VOHC). Jitendra Kumar Chaudhary and colleagues proposed that therapeutic targeting with drugs acting on the host and the host–virus interface might offer advantages in terms of specificity, efficiency and durable responses; as such, targets are less likely to undergo mutational resistance [17].

In summary, this Special Issue is fully dedicated to addressing several aspects of COVID-19, including cellular inflammation and immunity. Overall, these contributions further strengthen the essential function of the immune system in health and diseases and reinforce the concept that the knowledge of immune pathways could hold great promise for fighting the pandemic.
